# The influence of perceived stress on the human microbiome

**DOI:** 10.1186/s13104-022-06066-4

**Published:** 2022-06-03

**Authors:** Austin T. Almand, Allison P. Anderson, Brianna D. Hitt, John C. Sitko, Rebekah M. Joy, Benjamin D. Easter, Erin A. Almand

**Affiliations:** 1 University of Colorado–Anschutz Medical Campus, Aurora, CO USA; 2 Department of Aerospace Engineering Sciences, University of Colorado, Boulder, CO USA; 3 Department of Mathematical Sciences, United States Air Force Academy, Colorado Springs, CO 80840 USA; 4 Department of Biology, United States Air Force Academy, Colorado Springs, CO 80840 USA

**Keywords:** Microbiome, Stress, Cortisol, 16S rRNA, Gut–brain axis

## Abstract

**Objective:**

Microbial dysbiosis, a shift from commensal to pathogenic microbiota, is often associated with mental health and the gut–brain axis, where dysbiosis in the gut may be linked to dysfunction in the brain. Many studies focus on dysbiosis induced by clinical events or traumatic incidents; however, many professions in austere or demanding environments may encounter continuously compounded stressors. This study seeks to explore the relationship between microbial populations and stress, both perceived and biochemical.

**Results:**

Eight individuals enrolled in the study to provide a longitudinal assessment of the impact of stress on gut health, with four individuals providing enough samples for analysis. Eleven core microbial genera were identified, although the relative abundance of these genera and other members of the microbial population shifted over time. Although our results indicate a potential relationship between perceived stress and microbial composition of the gut, no association with biochemical stress was observed. Increases in perceived stress seem to elucidate a change in potentially beneficial *Bacteroides*, with a loss in *Firmicutes* phyla. This shift occurred in multiple individuals, whereas using cortisol as a stress biomarker showed contradictory responses. These preliminary data provide a potential mechanism for gut monitoring, while identifying targets for downstream modulation.

## Introduction

Anxiety, depression and post-traumatic stress disorder remain a critical area of study for the well-being of individuals in high-stress environments, including our military servicemen and women. The prolonged conflicts and multiple deployments associated with United States military engagements have attracted attention to combat-related mental health issues [1, 2], but there are other at-risk populations, both within the military and the private sector (e.g., medicine, space flight, firefighting, polar region scientists, oil/gas extraction, etc.). Current research places an emphasis on acute medical conditions affecting immediate mission readiness [3–5]; however, preliminary inquiries into the gut-brain axis on chronic, but less acute, stress suggest studying this complex relationship may elucidate influencing factors on the mental health of individuals operating in high stress or austere environments [6].

While there are many studies evaluating the impact of a single event, or physical trauma, the ongoing, day-to-day stresses of a physically, mentally or emotionally rigorous job are often overlooked. An important baseline needs to be established for individuals exposed to chronic stress, and potential protective mechanisms should be studied to determine how the body deals with these perturbations normally, to identify potential interventions should the situation go awry.

This study sought to take a month-long stress and microbiome baseline of individuals, evaluate them during a week-long high stress event, and continue monitoring them for 1 month post exposure. However, 1 week prior to the beginning of the exercise in March 2020, the pre-planned high-stress event was canceled due to the Coronavirus Disease-19 pandemic, shifting the study from a predominantly physical stressor to a more mentally and emotionally stressful environment caused by pandemic isolation requirements. Baseline data were obtained from eight individuals in the United States prior to the first wave of the pandemic in February, with weekly sampling carried throughout the first wave of the pandemic, and in some cases, until May, according to the preference of the subject. While a deviation from the original plan, this study provides value by virtue of its timing: we were able to study mental health separate from physical stress and determine underlying microbial patterns in this perceived stress response.

## Main text

### Materials and methods

The study recruited participants from a convenience sample student cohort at the University of Colorado–Boulder. Investigators provided a lecture on the microbiome and the gut–brain axis, then at the conclusion of the lesson, informed the students about a study in which they could participate in a voluntary manner. Interested participants had 2 weeks to enroll and were consented by a member of the research team not involved in classroom instruction. Once consented, individuals received a sampling packet with instructions on tasks to complete at various intervals: upon enrollment (Initial General Survey), monthly (Outcome Questionnaire-45, Insomnia Severity Index Health Questionnaire, Patient Health Questionnaire-8) and weekly (Weekly General Survey, Perceived Stress Scale, personal samples), with access to the surveys electronically. There were two different sample categories collected: microbiome and saliva. The microbiome samples were obtained by vigorously rubbing a BD BBL^™^ CultureSwab^™^ EZII (East Rutherford, New Jersey, USA) on either the palm (a more transient microbial population), forearm (relatively stable), or on used toilet paper (indicative of the gut microbiome). Participants provided saliva samples using a Salivette^™^ (Sartstedt, Nümbrecht, Germany), following manufacturer’s instructions. Individuals collected their own samples at approximately the same time every day and were instructed to deposit them in a specific −80 °C freezer for further processing by the investigative team.

Once all samples were collected, investigators scored the surveys according to published guidelines [7, 8]. Microbiome samples underwent DNA extraction using the Qiagen QiAmp Fast DNA Stool Mini Kit (Venlo, Netherlands). Samples were mailed, overnight on ice, to the Genewiz processing facility (South Planfield, New Jersey) for 16S rRNA amplicon sequencing, followed by bioinformatics and statistical analyses through their 16S-EZ pipeline. Briefly, a multiplexed primer set amplified the V3 and V4 hypervariable region of the 16S rRNA gene, prior to 2 × 250 bp Illumina sequencing. Chimera sequences were removed using UCHIME ‘Gold’ database and clustered by operational taxonomic units and mapped using the Nt database to identify the community composition. Saliva samples were processed using the Cortisol Enzyme Linked Immunosorbent Assay kit (Enzo, Farmingdale, New York, USA), according to manufacturer’s instructions. Once all data was collected, it was cleaned and formatted using RStudio.

### Results and discussion

**Fig.1:**
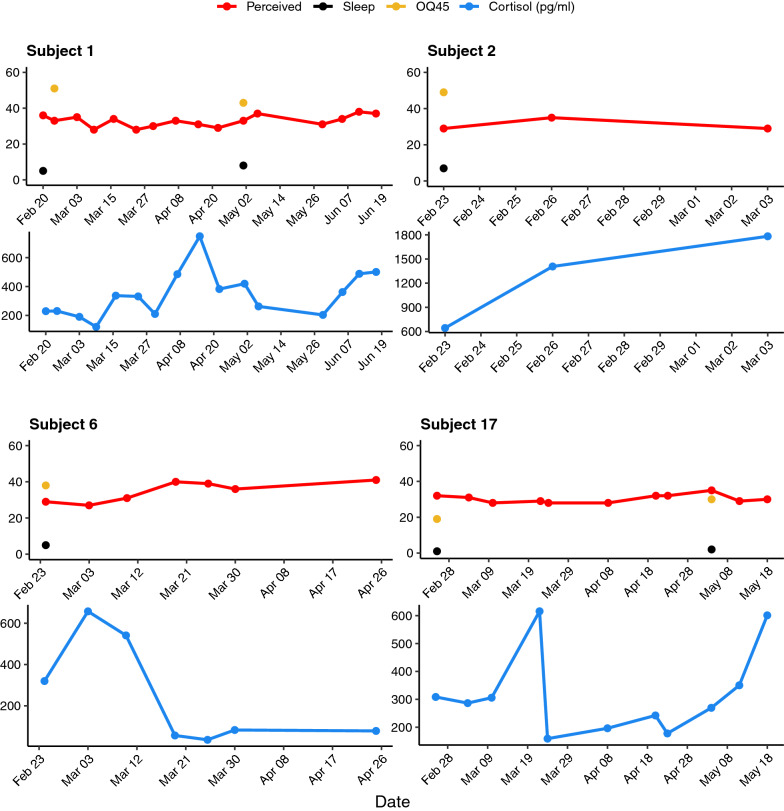
Perceived and biochemical stress of each participant over time. The cortisol (blue) and perceived stress scale (red) were taken weekly, whereas the sleep score (black) and OQ−45 (gold) were taken bimonthly. The maximum and minimum points generated from these data were used to choose time points of interest for further microbial analysis

Individual cortisol levels were plotted against their corresponding survey data, to determine if there was a relationship between the perceived stress, measured via surveys, and the biochemical response to stress, measured via cortisol. From this preliminary comparison, it appears in many instances a high level of perceived stress did not translate to a high level of biochemical stress, as the high and low values for each of these measurements happened on different days, often drastically separated over time (Fig. 1). This discordance is likely due to variation across the group’s sampling methodology and sampling disparities on the individual level since cortisol cycles throughout the day [9, 10], making repetition and consistency important factors. Conversely, the survey data, while variable and subjective, provided a more consistent evaluation of an individual’s perceived stress level.

**Fig.2:**
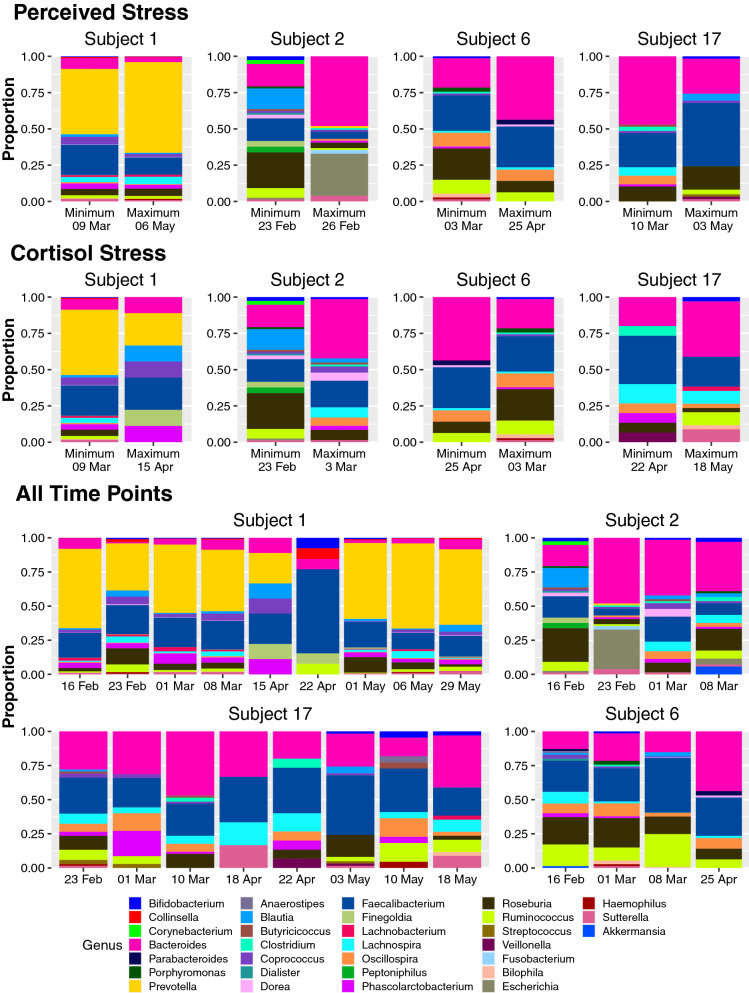
Diversity of the gut microbiome for each participant over time. Gut microbiome diversity is displayed for the time points corresponding to the minimum and maximum perceived stress levels (top panel), the time points corresponding to the minimum and maximum cortisol levels (middle panel), and all time points measured (bottom panel). In the instances where the time points corresponding to the minimum or maximum did not yield high quality microbiome data, the time point with the next lowest (for the minimum) or next highest (for the maximum) perceived stress or cortisol levels were used.

For each subject, researchers identified the maximum and minimum values for both the cortisol measurements and survey data, then compared the 16S rRNA metagenomics data from the same days, or in the instances where those days did not yield high quality microbiome data, from the days corresponding to the next highest (for the maximum) or next lowest (for the minimum) cortisol or survey values (Fig. 2 a–d). This evaluation sought to compare the days which should potentially have the highest variation to determine if there were underlying patterns in gut microbiota corresponding with perceived or biochemical stress. For the perceived stress, moving from the minimum value to the maximum value, there was a shift [Shannon diversity mean difference of 0.3458, with a 15.41% decrease and a Simpson evenness mean difference of 0.0892, with a 10.69% (Additional Data File)] from a more diverse population to a less diverse population that may be compensated by an increase in immunomodulatory microbes such as *Bacteroides*, *Streptococcus* and *Veillonella* [11, 12]. This increase in genera often associated with improved gut health suggests that perception of stress is potentially tied to a biochemical pathway capable of modulating gut microbiota to ensure gut health despite adverse circumstances.

Conversely, there is no clear picture of a microbial response when examining gut microbiota corresponding to the maximum and minimum cortisol levels. Instead, there are contradictory responses: one response is a decrease in *Bacteroides* and increase in *Firmicutes*, leading to an overall decrease in diversity (Fig. 2e–g) and the other response is an increase in *Bacteroides* and decrease in *Firmicutes* with an overall shift towards increased diversity (Fig. 2h). For those with a shift in diversity, the shifts varied from a strong decrease (1.26 difference, 63.38% decrease in Shannon diversity) to a strong increase (0.6217 difference, 64.45% increase in Shannon diversity). This lack of a clear pattern may have several causes. First, the cyclic nature of cortisol may have inflated the stress biomarker based on time of day, rather than the actual stress of the individual [9]. Additionally, studies on the response of the oral microbiome to cortisol and other stress hormones suggest increased stress may not elicit a change in population demographics but rather dynamics, ultimately impacting the metatranscriptomics of the microbiome, rather than the metagenomics [13]. Lastly, not all time points resulted in quality sequencing data, so these values may not be the true minimums or maximums observed, but rather the minimum and maximum with quality sequencing, making it more difficult to identify potential relationships.

Interestingly, across the four individuals that provided multiple time points for this study, there was a core microbiome of 11 genera that emerged and varied throughout the group. Despite this uniformity in composition, each individual’s microbiome retained personal variability, fluctuating on the individual level over time (Fig. 2 h–l). These microbes, identified as the genera: *Bifidobacterium*, *Bacteroides*, *Blautia*, *Coprococcus*, *Faecalibacterium*, *Lachnospira*, *Oscillospira*, *Phascolarctobacterium*, *Roseburia*, *Ruminococcus*, and *Sutterella*, are commonly associated with gut health, and many are marketed as probiotics [14–16].

### Conclusions

This study explored the impact of perceived and biochemical stress on the human gut microbiome utilizing established mental health surveys, a known biomarker (cortisol), and 16S rRNA gene sequencing. It highlighted potential relationships between perceived stress and the gut microbiome, through modest but consistent shifts towards immunomodulatory bacteria in the presence of stress [11]. This study lays the framework and provides valuable insight for future inquiry into potential stress response microbial indicators.

## Limitations

- Inconsistency in participant-collected data. Due to the nature of the study, participants collected their own samples when convenient for them, and deposited them into a central freezer. This variation in collection time and technique likely resulted in the extreme swings in cortisol levels, as cortisol is known to cycle throughout the day. Additionally, many samples yielded poor quality DNA and sequencing data, limiting the relevant comparisons.
- Small study size. The initial study design incorporated a field exercise, limiting the potential participants to 20. Of the 20 eligible, 8 signed up. With the shutdown of campuses and programs nationwide, only four individuals provided three or more samples, and some of the samples ended up being mailed to the investigators, adding to the inconsistencies in the data.
- Study design. The original study centered around variations in microbiome, cortisol and perceived stress in relation to a physically and mentally taxing field exercise. The time points during the exercise would have been compared to the baseline set up via samples for 3 weeks before and a potential return to baseline, with sampling 3 weeks after the conclusion of the exercise. This set up would have allowed us to maintain consistency in sampling during the controlled middle portion, conduct multiple additional training sessions and keep better control of the samples to reduce storage variability. Additionally, it would have prompted better avenues for inquiry, with multiple time points before, during and after a known stressful event, versus the varying stress encountered by individuals adjusting to life in a global pandemic.

## Data Availability

The datasets generated and analyzed during this study are included in the supplementary data or from the corresponding author on reasonable request.
